# Functional brain imaging using 18F-fluorodeoxyglucose positron emission tomography/computerized tomography in 138 patients with Kleine–Levin syndrome: an early marker?

**DOI:** 10.1093/braincomms/fcab130

**Published:** 2021-06-17

**Authors:** Benjamin Dudoignon, Laure-Eugénie Tainturier, Pauline Dodet, Géraldine Bera, Elisabeth Groos, Charlotte Chaumereuil, Jean-Baptiste Maranci, Aurélie Kas, Isabelle Arnulf

**Affiliations:** 1 Sorbonne University, Paris Brain Institute (ICM), Inserm UMR-S975, CNRS UMR7225, Paris 75013, France; 2 Sleep Disorders Unit, National Reference Centre for Kleine-Levin Syndrome, Pitié Salpêtrière University Hospital, APHP, Paris 75013, France; 3 Nuclear Medicine Department, Hôpitaux Universitaires Pitié-Salpêtrière University Hospital, Sorbonne University, AP-HP, Paris 75013, France; 4 LIB, INSERM U1146, 75013 Paris, France

**Keywords:** Kleine–Levin syndrome, FDG-PET/CT, functional imaging, adolescence, cognition

## Abstract

Kleine–Levin syndrome is a rare disorder characterized by relapsing-remitting episodes of severe hypersomnia, cognitive impairment, apathy, derealization and behavioural disturbances. Between episodes, most patients experience normal sleep, mood and behaviour, but they may have some residual abnormalities in brain functional imaging; however, the frequency, localization and significance of abnormal imaging are unknown, as brain functional imaging have been scarce and heterogenous [including scintigraphy 18F-fluorodeoxyglucose positron emission tomography/computerized tomography (FDG-PET/CT) and functional MRI during resting state and cognitive effort] and based on case reports or on group analysis in small groups. Using visual individual analysis of 18F-fluorodeoxyglucose positron emission tomography/computerized tomography at the time of Kleine–Levin syndrome diagnosis, we examined the frequency, localization and clinical determinants of hypo- and hypermetabolism in a cross-sectional study. Among 179 patients with Kleine–Levin syndrome who underwent 18F-fluorodeoxyglucose positron emission tomography/computerized tomography, the visual analysis was restricted to the 138 untreated patients studied during asymptomatic periods. As many as 70% of patients had hypometabolism, mostly affecting the posterior associative cortex and the hippocampus. Hypometabolism was associated with younger age, recent (<3** **years) disease course and a higher number of episodes during the preceding year. The hypometabolism was more extensive (from the left temporo-occipital junction to the entire homolateral and then the bilateral posterior associative cortex) at the beginning of the disorder. In contrast, there was hypermetabolism in the prefrontal dorsolateral cortex in half of the patients (almost all having concomitant hypometabolism in the posterior areas), which was also associated with younger age and shorter disease course. The cognitive performances (including episodic memory) were similar in patients with versus without hippocampus hypometabolism. In conclusion, hypometabolism is frequently observed upon individual visual analysis of 18F-fluorodeoxyglucose positron emission tomography/computerized tomography during asymptomatic Kleine–Levin syndrome periods; it is mostly affecting the posterior associative cortex and the hippocampus and is mostly in young patients with recent-onset disease. Hypometabolism provides a trait marker during the first years of Kleine–Levin syndrome, which could help clinicians during the diagnosis process.

## Teaser

Kleine-Levin syndrome is a rare relapsing-remitting disease with no biological marker. Dudoignon et al. found that 70% of 138 patients had brain hypometabolism in FDG-PET/CT during remitting periods, affecting the posterior associative cortex and the hippocampus. This hypometabolism provides a trait marker for diagnosis.

## Graphical Abstract

**Graphical Abstract:**
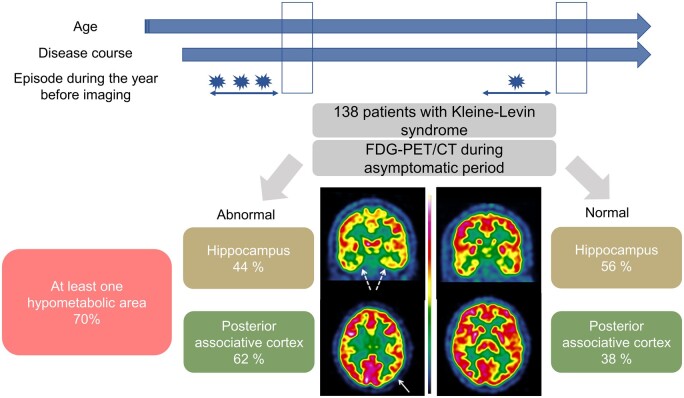

## Introduction

Kleine–Levin syndrome (KLS) is a rare (3/million) neurological disorder affecting mostly teenagers or young adults. The syndrome is characterized by recurrent, relapsing-remitting episodes of severe hypersomnia, cognitive impairment, apathy and derealization, sometimes associated with disinhibited behaviour (megaphagia, hypersexuality and rudeness), altered mood and hallucinations.^1^ Sleep, mood, cognition and behaviour are usually normal between episodes, although some mild cognitive abnormalities may persist during asymptomatic periods,^2^^,^^3^ as well as emerging psychiatric disorders in 20% of patients.^4^ The mechanisms and causes of KLS are unknown, but autoimmune or inflammatory hypotheses are suggested. The diagnosis is clinical, as there is yet no identified marker in the cerebrospinal fluid, and morphological brain imaging is normal.^5^ Consequently, the diagnosis is easy in the presence of clear-cut brief (e.g. 1 week) episodes with severe symptoms in adolescents, but it is more difficult in cases with unsure or absent history, amnesia, long episodes, attenuated or unusual symptoms and concomitant psychiatric or neurological disorders.

Of interest, functional brain imaging may be abnormal during symptomatic and asymptomatic periods. Case reports and small series have identified some hypoperfusions in single photon emission computed tomography (SPECT) using ethyl cysteinate dimer (ECD) or hexa-methyl-propryl-amineoxime (HMPAO) and, more recently, some hypometabolisms using 18F-fluorodeoxyglucose positron emission tomography/computerized tomography (FDG-PET/CT).^6^ During episodes, the hypoperfused area includes the hypothalamus,^7^ thalamus,^7–9^ basal ganglia^8^ and some cortical areas in parts of the frontal,^9^^,^^10^ temporal^9^^,^^11^^,^^12^ and parietal^9^ cortex. Group analysis using ECD SPECT in 11 patients showed that, compared to asymptomatic periods, there was hypoperfusion in the right dorsomedial prefrontal cortex and the right parieto-temporal junction.^13^ In the FDG-PET/CT, hypometabolism is also found in the hypothalamus, in both caudate nuclei, and in the striatum in single cases.^14^^,^^15^ Decreased metabolism in the occipital and temporal gyrus (versus para-central, pre-central and post-central areas, supplementary motor area, medial frontal gyrus, the thalamus and putamen hypermetabolism) was found in four patients during symptomatic versus asymptomatic periods.^16^

In addition, in case reports, some local hypoperfusions were found to possibly persist during the asymptomatic period.^9^^,^^11^ The thalamus metabolism was initially targeted as a specific marker of KLS, as it was decreased on ECT SPECT during episodes in 7/7 children with KLS (and normalized during asymptomatic periods) on one hand^9^ and was recruited in KLS patients (but not in controls) during a memory task using fMRI and magnetic resonnance (MR) spectroscopy in asymptomatic periods.^17–19^ However, other regions (mostly in the cortex) than the thalamus were abnormal using brain functional imaging. Compared to healthy controls using group analysis, 41 patients presented during asymptomatic periods hypoperfusions on ECD SPECT in the thalamus, hypothalamus and caudate nucleus and in cortical areas, including the orbitofrontal cortex, anterior cingulate cortex and left superior temporal gyrus, extending to the insula.^13^ On visual analysis, 11 of 24 KLS patients had hypoperfusion using HMPAO SPECT in the temporal or frontotemporal regions.^20^ FDG-PET has been visually analysed during asymptomatic periods in a single case report showing mild residual hypometabolism in the cingulate gyri, temporal lobes, left frontal lobe, left parietal lobe and right parahippocampus.^21^ Compared to healthy controls using group analysis, four patients with KLS had no hypometabolism but hypermetabolism in the frontal cortex, inferior and medial temporal gyri, left posterior cingulate and right precuneus.^16^

Overall, this information about brain functional imaging is scarce in KLS and is mostly based on case reports or on group analysis in groups of 4 to 41 patients. Additionally, it is difficult to perform functional imaging during symptomatic episodes (in agitated, uncompliant patients). Eventually, most patients are referred to reference centres during asymptomatic periods when they are freely willing to undergo the FDG-PET/CT. As the FDG-PET/CT has been a routine test in our centre since 2012, we decided to collect retrospectively the FDG-PET/CT information obtained during asymptomatic periods at the time of diagnosis in a large series of well-characterized patients with KLS. Our aim was to provide some useful clues for clinicians, including reporting on the sensitivity of the test, the common cerebral locations where hypo- and hypermetabolism should be looked for, and their demographic and clinical determinants.

## Materials and methods

### Subjects

The study was performed in the National Reference Centre for KLS in the Pitie-Salp**ê**tri**è**re University Hospital in Paris between December 2011 and March 2019. All patients referred for suspected KLS and their families underwent a long interview with the KLS physicians (including a neurologist and a psychiatrist, both sleep specialists), a cognitive assessment and blood sampling. The physicians examined previous medical information collected before referral, including medical history, brain morphological imagery, EEG, sleep studies, the results of the spinal tap if performed, as well as biological results (mostly autoantibodies and urine organic acid chromatography). To be included in the present study, patients had to meet the international KLS criteria,^22^ including the following: (i) experiencing at least two episodes of excessive sleepiness and sleep duration, each persisting for 2 days to 5 weeks; (ii) episodes recur usually more than once a year and at least once every 18** **months; (iii) the patient has normal alertness, cognitive function, behaviour and mood between episodes; (iv) the patient must demonstrate at least one of the following during an episode: (1) Cognitive dysfunction; (2) Altered perception; (3) Eating disorder (anorexia or hyperphagia); (4) Disinhibited behaviour (such as hypersexuality); and (v) symptoms are not better explained by another sleep, medical, neurologic or psychiatric disorder (especially bipolar disorder), or use of drugs or medications. Patients with atypical or differential diagnoses, those with KLS secondary to other disorders and those with severe mental deficiency were excluded. Notably, this cohort of KLS patients was different from the 41 patients with KLS who benefitted from SPECT analysis between 2007 and 2011.^13^ For this analysis, patients who had the FDG-PET/CT during an episode were excluded, as well as those already treated for KLS at the time of the scan. All patients (including minor patients, in this case, in association with their parent-signed consent) signed an informed consent form for having their measures collected in the study. The research programme was approved by the ethics committee.

### Clinical measures and cognitive function

The patients completed the Stanford KLS questionnaire,^23^ which was later reviewed face to face with the neurologist. In addition, we paid attention to the last year of the disorder (just before brain imaging) and measured the number and mean duration of episodes, the time incapacitated during the last year and the time since the last episode. Patients completed the Depersonalization/Derealization Inventory regarding symptomatic periods^24^ and the apathy scale during symptomatic and asymptomatic periods.^25^ Physicians performed a physical examination. Regarding cognitive function during asymptomatic periods, the patients underwent cognitive tests the same day as the functional brain imaging, as previously described.^3^

### Positron emission tomography

Dedicated 3D brain FDG-PET/CT was acquired using Gemini Dual PET/CT (Philips Medical Systems) 30** **min after the injection of 2 MBq/kg FDG (range: 125–250 MBq). Patients fasted for at least 4** **h with glycaemia <1.4** **g/l. They rested in quiet surroundings with the eyes closed at least 20-_** **_min post-injection. The FDG-PET/CT acquisition lasted 15 min with inline CT for attenuation correction. A conventional 3D iterative algorithm was used to reconstruct images, including attenuation, scatter and random coincidence corrections, and a post-reconstruction filter in a 128 × 128 matrix. Qualitative analysis of the FDG-PET/CT was performed by one board of two nuclear medicine physicians with 6 and 11 years of experience in neuroimaging, respectively (G.B. and A.K.). The age and sex of the patients at the time of the examination were the only information available during reading sessions (these physicians ignored whether the patient had a true diagnosis of KLS, was in symptomatic or asymptomatic episodes and was or was not treated). Visual interpretation was performed with the Advantage Workstation (ADW 4.7, GE Healthcare) with images reoriented on the intercommissural line and displayed in three orthogonal views with the French colour scale. Activity normalization was performed by setting the maximum activity on the pons. The whole brain was examined. In particular, regional cerebral glucose metabolism was scored as normal, hypometabolic or hypermetabolic in the following regions: the associative posterior cortex (including the lateral parietal, occipital, temporal areas, precuneus and temporo-occipital junction), the prefrontal cortex (orbitofrontal, mediofrontal and dorsolateral areas), the hippocampus, striatum, thalamus and cerebellum. In a second step, the conclusion of the FDG-PET/CT visual interpretation (normal versus abnormal) was compared to the medical report produced in clinical practice at the time of the FDG-PET/CT examination to assess concordance between readings using Cohen’s kappa test. The inter-scorer agreement was 0.80. To take into account the extent of anomalies in the posterior cortex, a second classification was performed identifying five cortical patterns: (i) normal metabolism, (ii) mild hypometabolism involving only the left temporo-occipital junction, (iii) hypometabolism involving the whole associative posterior cortex of the left hemisphere, (iv) hypometabolism involving the right-associative cortex without anomalies on the left side and (v) hypometabolism of the bilateral posterior associative cortex.

### Statistical analysis

Qualitative measures are described using percentages, and quantitative measures are described by means and standard deviations. Comparisons between groups were performed using chi-square or Fisher tests for qualitative measures and Student’s *t*-test for quantitative measures. Ordinal logistic regression was performed with the type of the hypometabolism as the ordinal qualitative dependant variable and the clinical quantitative parameters as a regressor.

### Data availability

Detailed data are available upon request, provided that the requested clinical measures cannot lead to breach the patient anonymity.

## Results

### Clinical characteristics of the patients

**Figure 1:**
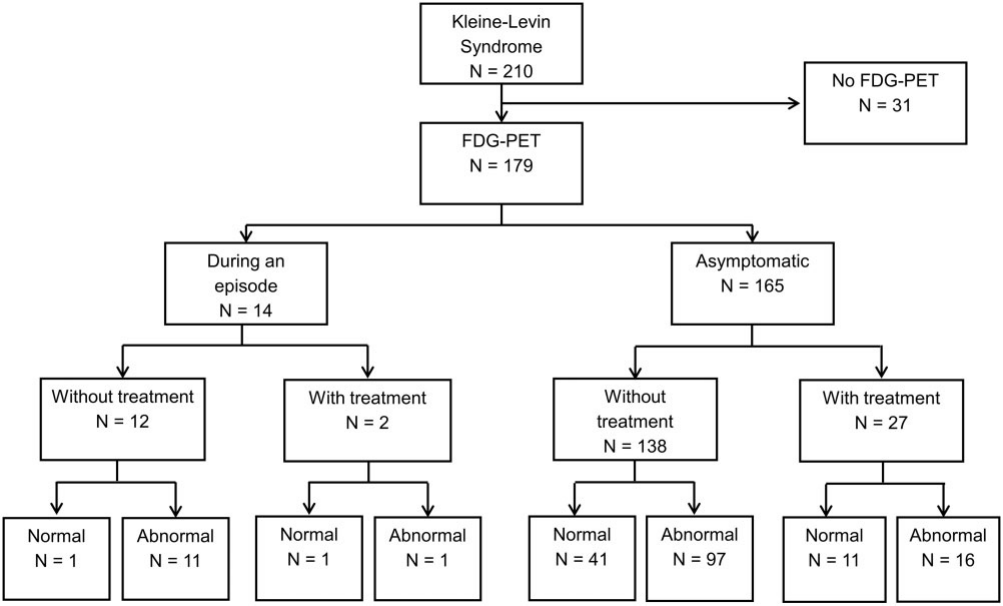
Flow chart of patients with KLS and frequency of abnormal (with hypometabolism) FDG-PET/CT.

Between December 2011 and March 2019, ∼260 patients suspected to have KLS were referred to the expert centre. Among them, 21 were excluded because they had no KLS. Atypical cases were also excluded. Eventually, 210 patients had clear-cut KLS (Fig. 1). Thirty-one patients did not have the FDG-PET/CT for medical or logistical reasons (non-availability or technical problems of the FDG-PET/CT system, unavailable pregnancy test for some women, and one case with diabetes). Among 179 patients who underwent the FDG-PET/CT, 14 patients underwent a scan during an episode (of whom 2 were treated), and 27 patients were asymptomatic but had already received preventive treatment for KLS (valproate or lithium). Twelve (85.7%) of the 14 patients who were scanned during an episode also had abnormal FDG-PET/CT. This percentage was not different from the percentage (70.3%) of 138 untreated patients with abnormal FDG-PET/CT during the asymptomatic period (*P* = 0.23). Sixteen (59%) of the 27 patients who were scanned during an asymptomatic period but were taking medication also had abnormal FDG-PET/CT, a percentage that was not different (*P* = 0.26) from that of the untreated asymptomatic group.

Eventually, the 138 patients with KLS who were asymptomatic and did not receive any treatment at the time of the FDG-PET/CT scan were included in the present analysis. Their demographic and clinical characteristics are shown in Table 1. During at least one of the episodes, 138 (100%) patients had hypersomnia, 138 (100%) had cognitive impairment, 137 (99%) had apathy, 133 (96%) had derealization, 88 (64%) had disinhibited behaviour [including megaphagia in 49 (36%) and hypersexuality in 32 (24%)], 54 (39%) had mood disturbances, 40 (29%) had hallucinations or delusions and 37 (27%) had hyporexia.

**Table 1:** Demographic and clinical characteristics of patients with KLS, in the function of FDG-PET/CT results

	Total group	Hypometabolism in the posterior associative cortex			Hippocampus hypometabolism			Prefrontal dorsolateral hypermetabolism		
		Without	With	*P*	Without	With	*P*	Without	With	*P*
No of patients	138	52	86		77	61		90	48	
Demographical characteristics										
Sex, % female	34.8 (48)	23.1 (12)	41.9 (36)	**0.025**	35.1 (27)	34.4 (21)	0.94	30 (27)	43.8 (21)	0.08
Birth problems, %	16.7 (23)	15.4 (8)	17.4 (15)	0.75	10.4 (8)	24.6 (15)	**0.026**	16.7 (15)	16.7 (8)	1.00
Left-handed or ambidextrous, %	17.4 (24)	21.2 (11)	15.1 (13)		19.5 (15)	14.8 (9)	0.47	18.9 (17)	14.6 (7)	0.53
Clinical measures										
Age at disease onset, y	16.3** ± **3.7	16.4** ± **3.8	16.2** ± **3.7	0.73	16.8** ± **4.2	15.6** ± **3.0	0.06	16.2** ± **3.5	16.4** ± **4.3	0.79
Age at FDG-PET/CT time, y	21.6** ± **9.2	24.9** ± **12.2	19.6** ± **6.0	**0.004**	23.9** ± **11.0	18.8** ± **5.0	**<0.001**	22.6** ± **10.4	19.8** ± **6.0	**0.046**
Number of days of the first episodes, days	15.1** ± **19.6	13.9** ± **10.5	15.9** ± **23.5	0.51	16.0** ± **20.4	14.0** ± **18.6	0.56	15.3** ± **16.6	14.9** ± **24.4	0.93
Time since last episode, days	98.8** ± **75.8	103.8** ± **75.2	95.7** ± **76.4	0.46	95.8** ± **72.5	102.5** ± **80.2	0.88	95.8** ± **72.9	104.3** ± **81.4	0.55
Disease course, month	60.5** ± **91.3	94.7** ± **130.3	40.4** ± **47.9	**0.006**	78.8** ± **112.3	37.0** ± **44.1	**0.004**	72.1** ± **108.6	39.6** ± **38.8	**0.013**
Since disease onset										
Number of episodes	14.6** ± **31.9	20.8** ± **48.8	10.8** ± **13.2	0.15	18.7** ± **41.6	9.3** ± **8.8	0.06	15.7** ± **38.0	12.3** ± **14.9	0.46
Time incapacitated, d	119.7** ± **139.3	129.1** ± **138.5	114.4** ± **140.3	0.52	146.6** ± **173.6	88.6** ± **74.0	**0.015**	120.5** ± **140.5	118.2** ± **138.5	0.93
Mean episode duration, d	13.1** ± **13.4	13.2** ± **11.1	13.1** ± **14.6	0.94	13.4** ± **9.7	12.9** ± **16.7	0.91	13.4** ± **11.5	12.8** ± **16.7	0.87
Year before FDG-PET/CT										
Number of episodes	3.4** ± **2.8	2.8** ± **2.6	3.8** ± **2.9	**0.037**	2.7** ± **2.2	4.3** ± **3.2	**0.001**	3.1** ± **2.5	4.0** ± **3.2	0.07
Time incapacitated, d	40.4** ± **33.3	37.9** ± **35.8	41.8** ± **31.9	0.53	39.2** ± **32.9	41.9** ± **34.0	0.65	38.1** ± **30.5	44.5** ± **38.0	0.33
Mean episode duration, d	18.9** ± **25.9	23.5** ± **35.1	16.4** ± **19.2	0.21	22.1** ± **29.7	14.7** ± **19.6	0.10	19.9** ± **25.9	17.0** ± **26.2	0.55

The bold text corresponds to the significant results.

### Brain hypometabolism on the FDG-PET/CT

**Figure 2:**
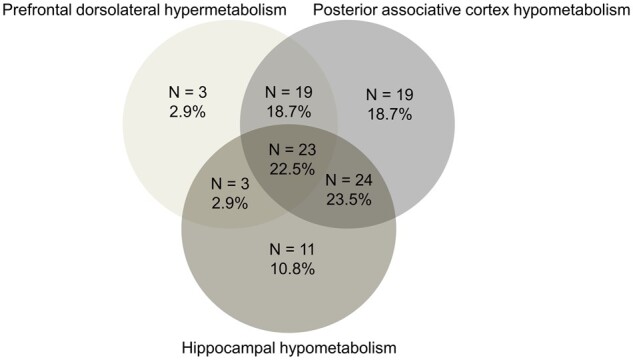
Associations between areas with FDG-PET/CT hypometabolism in patients with KLS.

**Figure 3:**
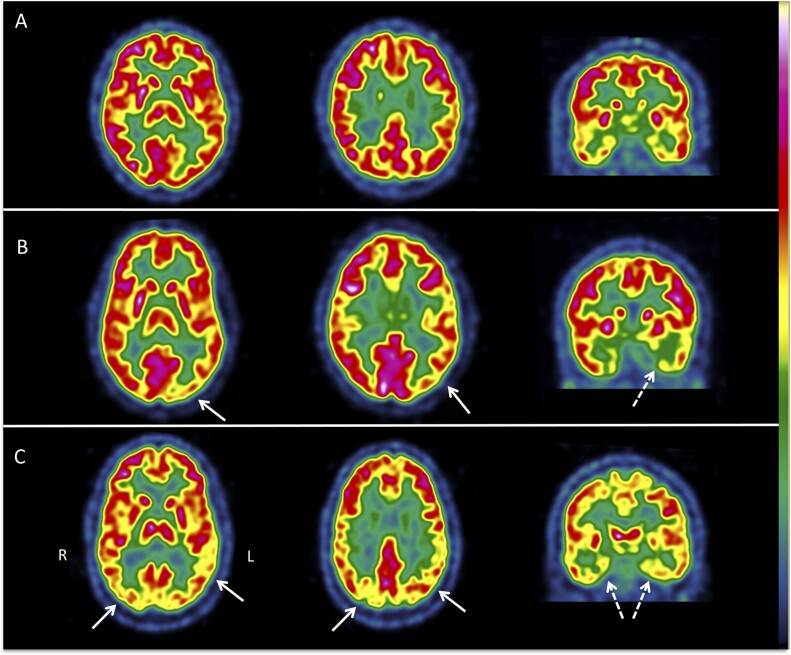
**Example of brain FDG-PET/CT images in three patients with KLS.** The images are displayed in axial and coronal views in radiological convention [right (R) is on the left (L)] with the French colour scale. Patient A, 18 y.o., had two episodes with hypersomnia, derealization, apathy and mood disturbances before the FDG-PET/CT. The cortical and subcortical metabolism was normal. Patient B, 17 y.o., had six episodes with hypersomnia, derealization, apathy, behavioural disturbances, hallucinations and psychiatric symptoms before the FDG-PET/CT scan. There was mild left temporo-occipital (white arrow) and hippocampal hypometabolisms (white dotted arrow). Patient C, 36 y.o., had 21 episodes with hypersomnia, derealization, apathy, behavioural disturbances and psychiatric symptoms. There was a bilateral associative parieto-temporo-occipital (white arrow) and hippocampal hypometabolism (white dotted arrow).

There were at least one or more hypometabolic areas (designed as ‘abnormal FDG-PET/CT’) in 97/138 (70.3%) untreated patients scanned during asymptomatic periods (Table 2). The most frequent abnormalities were localized in the posterior associative area (61.6%) and in the medial temporal regions (44.2%). In contrast, only 6.5% of patients had thalamic hypometabolism, and 2.9% had cerebellar hypometabolism. No hypometabolism was found in the prefrontal cortex. The left hemisphere was more often affected than the right hemisphere. The frequencies of bilateral, left or right hypometabolisms in the hippocampus and the posterior associative cortex were not different between left/ambidextrous and right-handed patients (data not shown). The combination of hypometabolism and hypermetabolism in the same patients is shown in Fig. 2. All but three patients with hypermetabolism had concomitant hypometabolism in the posterior associative cortex. Among the 85 patients with hypometabolism in the posterior associative cortex, 24 (28.2%) patients had hypometabolism limited to the temporo-occipital junction, 41 (48.2%) had hypometabolism of the left-associative parieto-temporo-occipital cortex and 20 (23.5%) had hypometabolism in the bilateral posterior associative cortex (examples in Fig. 3).

**Table 2:** Abnormalities in the FDG-PET/CT in 138 patients with KLS

Abnormalities	% (N)
Hypermetabolism	
Prefrontal dorsolateral	34.8 (48)
Left only	0.0 (0)
Right only	29.7 (41)
Bilateral	5.1 (7)
Precuneus	11.6 (16)
Hypometabolism	
Posterior associative area	
Temporo-occipital junction	17.4 (24)
Left only	16.0 (22)
Right only	1.4 (2)
Bilateral	0.0 (0)
Temporo-parieto-occipital cortex	44.2 (61)
Left only	29.7 (41)
Right only	0.0 (0)
Bilateral	14.5 (20)
Other areas	
Hippocampus	44.2 (61)
Left only	16.0 (22)
Right only	6.5 (9)
Bilateral	21.7 (30)
Thalamus	6.5 (9)
Right only	0.0 (0)
Bilateral	1.4 (2)
	5.1 (7)
Cerebellum	2.9 (4)
Left only	0.0 (0)
Right only	0.0 (0)
Bilateral	2.9 (4)

FDG-PET/CT = 18F-fluorodeoxyglucose positron emission tomography/computerized scan.

### Factors associated with hypometabolism

**Figure 4:**
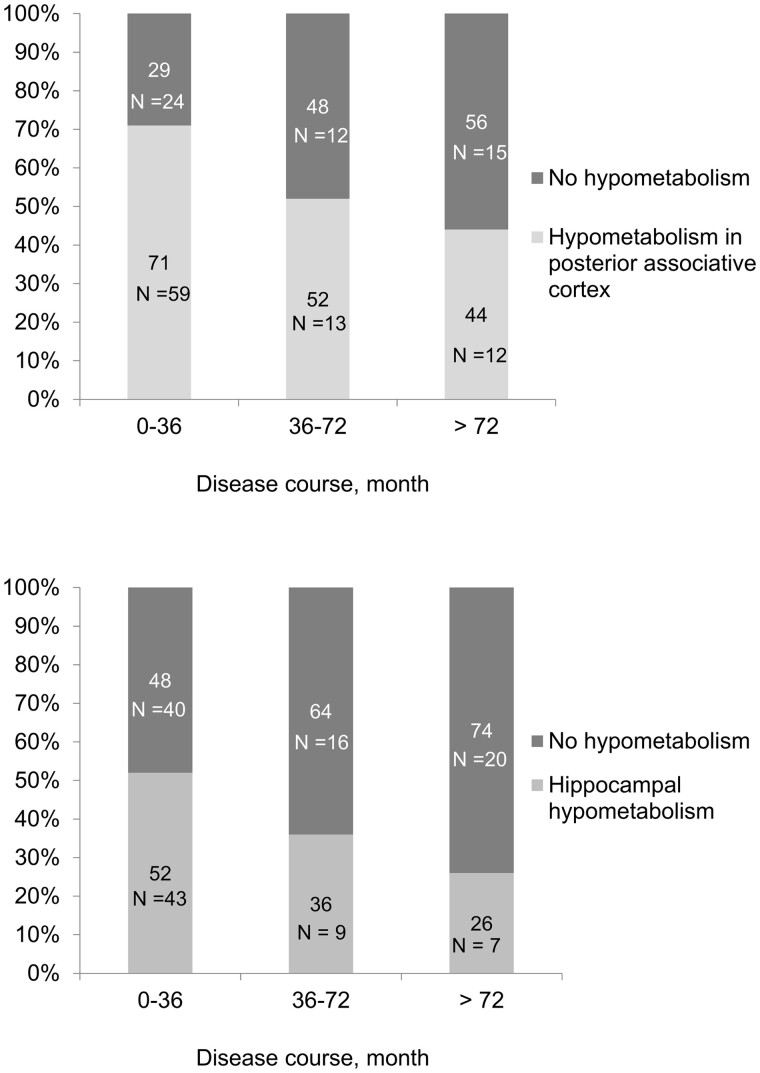
**Patients with FDG-PET/CT hypometabolism according to disease course.** Percentage of patients with FDG-PET/CT hypometabolism in the posterior associative area (upper panel) and mesotemporal structure (lower panel) according to disease course. Patients with imprecise disease course are omitted.

The factors associated with at least one hypometabolism in the posterior associative cortex included the female sex, a younger age at the time of the FDG-PET/CT, a shorter disease course and a higher number of episodes during the preceding year (Table 1). The frequency of patients with hypometabolism in this area, depending on the disease course, is illustrated in Fig. 4. Within the posterior associative cortex, more severe hypometabolism was associated with a progressively shorter disease course, a lower number of episodes (measured over the total disease course) and a progressively shorter amount of time spent incapacitated (Table 3). Hypometabolism in the hippocampus was associated with more frequent birth defects, younger age, shorter disease course, a shorter amount of time spent incapacitated (over the total disease course) and a higher number of episodes during the preceding year. The recency of the episode was not responsible for this result, as the frequencies of hypometabolism in the posterior associative cortex and in the hippocampus, as well as of the frontal hypermetabolism in patients with versus without recent (<2 months) episodes were similar (data not shown). Because the hippocampus is involved in memory processes, we evaluated whether cognitive impairments (measured during the cognitive assessment performed the same day as the FDG-PET) correlated with hypometabolism in this structure in an exploratory study (Table 4). The performances on the verbal episodic memory test, spatial memory test, and executive and attentional function tests were similar in patients with and without hippocampal hypometabolism. However, there were more patients with working memory impairment (measured as performance on the backward and forward digit tests) in the group with hippocampal hypometabolism than in the group without hippocampal hypometabolism.

**Table 3:** Demographical and clinical characteristics depending on the extension of the hypometabolisms within the posterior associative cortex

Hypometabolism	Temporo- occipital junction	Left posterior associative cortex	Bilateral	*P*
			posterior associative cortex	
Number of patients	24	41	20	
Sex, % male (N)	62.5 (15)	58.5 (24)	55 (11)	0.61
Birth problems, % (N)	16.7 (4)	22 (9)	10 (2)	0.64
Clinical measures				
Age at disease onset, year	16.0 ± 2.6	16.2 ± 4.3	16.3 ± 3.9	0.77
Age at FDG-PET/CT, year	21.0 ± 7.4	19.5 ± 5.6	18.2 ± 5.1	0.12
First episode duration, day	19.5 ± 35.9	17.3 ± 19.5	8.8 ± 5.9	0.16
Time since last episode, day	78.4 ± 56.7	106.4 ± 75.8	82.9 ± 84.6	0.56
Disease course, month	62.2 ± 72.9	37.4 ± 32.8	20.6 ± 21.2	**0.004**
Events since disease onset				
Number of episodes	17.5 ± 20.5	8.2 ± 8.5	8.3 ± 6.0	**0.018**
Time incapacitated, day	162.1 ± 199.8	102.0 ± 115.0	67.1 ± 47.2	**0.04**
Mean episodes duration, day	11.3 ± 10.4	13.6 ± 9.3	9.2 ± 8.7	0.85
Events during the year preceding the FDG-PET/CT				
Number of episodes	3.9 ± 2.4	3.0 ± 2.6	5.3 ± 10.4	0.16
Time incapacitated, day	42.7 ± 22.7	37.0 ± 32.2	46.4 ± 36.8	0.76
Mean episode duration, day	17.4 ± 20.9	15.6 ± 13.4	11.4 ± 10.7	0.20

The *P*-value is the result of ordinal logistic regression with the type of hypometabolism classified in the following order: (i) temporo-occipital junction; (ii) left posterior associative cortex; (iii) bilateral posterior associative cortex as independent variable and the clinical quantitative variable as regressor. The bold text corresponds to the significant results.

**Table 4:** Cognitive functions in patients with and without hippocampal hypometabolism

Cognitive domains	Hippocampal hypometabolism		
	Without	With	*P*
*N*	74	60	
Attention			
WMS digit span forward (0–9)	6.3 ± 1.1	6.7 ± 1.3	0.10
Subjects with deficient score (<5), % (*N*)	0 (0)	5 (3)	
Trail Making Test -A, sec	29.9 ± 15.3	29.1 ± 14.0	0.76
Subjects with deficient score, % (*N*)	4 (3)	10 (6)	
Stroop I, correct words	110.2 ± 17.7	108.7 ± 16.4	0.60
Subjects with deficient score, % (*N*)	11 (8)	10 (6)	
Stroop II, correct colour-words	100.4 ± 18.4	98.9 ± 17.1	0.63
Subjects with deficient score, % (*N*)	20 (15)	18 (11)	
Stroop III, correct colours	73.4 ± 12.1	71.5 ± 15.5	0.42
Subjects with deficient score, % (*N*)	5 (4)	5 (3)	
Working memory (Wechsler Memory Scale)			
Digit span backward (0–8)	4.6 ± 1.2	4.7 ± 1.2	0.42
Subject with deficit score (≤3 or delta >2 between forward and backward), % (*N*)	24 (18)	45 (27)*	0.012
Executive functions			
TMT-B, sec	64.5 ± 27.7	68.0 ± 39.5	0.57
Subjects with deficient score, % (*N*)	3 (2)	5 (3)	
Stroop Interference, correct words	46.2 ± 9.8	45.6 ± 11.3	0.74
Subjects with deficient score, % (*N*)	9 (7)	10 (6)	
Stroop Interference score	27.1 ± 7.9	25.7 ± 9.6	0.38
Subjects with deficient score, % (*N*)	3 (2)	7 (4)	
Semantic fluency (categories)	21.6 ± 6.1	22.0 ± 6.8	0.69
Subjects with deficient score, % (*N*)	20 (15)	5 (3)	
Verbal fluency (letter M)	12.2 ± 4.4	12.2 ± 4.4	0.93
Subjects with deficient score, % (*N*)	20 (15)	15 (9)	
Verbal fluency (letter P)	13.3 ± 4.8	13.1 ± 5.5	0.79
Subjects with deficient score, % (*N*)	27 (20)	33 (20)	
Episodic memory (Free and Cued Selective Reminding Test)			
Immediate free total recall (0–48)	34.1 ± 5.0	33.4 ± 5.4	0.41
Subjects with deficient score, % (*N*)	0 (0)	2 (1)	
Immediate total recall (0–48)	46.6 ± 1.6	46.0 ± 2.4	0.69
Subjects with deficient score, % (*N*)	11 (8)	18 (11)	
Delayed free recall (0–16)	12.8 ± 2.3	12.6 ± 2.4	0.69
Subjects with deficient score, % (*N*)	16 (12)	20 (12)	
Delayed cued recall (0–16)	15.7 ± 0.8	15.7 ± 0.6	0.75
Subjects with deficient score, % (*N*)	5 (4)	7 (4)	

### Regional hypermetabolism on the FDG-PET/CT

There was some regional hypermetabolism in 45 patients, including 3 without concomitant hypometabolism and 42 with concomitant hypometabolism (Table 2). Hypermetabolism was observed in the prefrontal dorsolateral cortex in 48 patients (41 with right dorsolateral cortex hypermetabolism and 7 with bilateral hypermetabolism) and in the precuneus in 16 patients (of whom 11 had concomitant prefrontal dorsolateral hypermetabolism), leading to 46% of KLS patients with hypermetabolism in the prefrontal dorsolateral and precuneus areas. No hypermetabolism was found in any other brain area. The disease course age at the FDG-PET/CT was lower in patients with hypermetabolism than in those without hypermetabolism (Table 1). No other demographical or clinical determinant of hypermetabolism was found. In particular, hypermetabolism was as frequent in patients with (23/48, 47.9%) megaphagia or hypersexuality (25/48, 52.1%, *P* = 0.44).

## Discussion

### Key findings

In this large series of 138 untreated patients with KLS, 70% of patients had hypometabolism visible in FDG-PET during an asymptomatic period. The most frequently affected structures included the posterior associative cortex and the hippocampus, whereas the thalamus and the cerebellum were rarely affected. Hypometabolism in the posterior associative cortex and in the hippocampus was associated with younger age, shorter disease course and a higher number of episodes during the preceding year (plus female sex for the posterior associative cortex). Hypometabolism was more extensive in the posterior associative cortex at the beginning of the disorder. In contrast, there was hypermetabolism in the prefrontal dorsolateral cortex and the precuneus in half of the patients (almost all having concomitant hypometabolism in the posterior structures), which was also associated with young age and shorter disease course.

### Brain hypometabolism is frequent during asymptomatic periods

Of interest, 70% of 138 untreated patients with KLS had visible hypometabolism on the FDG-PET/CT. Although several groups have previously studied brain functional imaging during asymptomatic KLS periods, methods (HMPAO and ECD SPECT, FDG-PET/CT and functional MRI) vary. Additionally, most studies are performed in single cases or small series.^6^ If one focuses on the FDG-PET/CT measures, only five patients with KLS (a series of four and an individual patient) have been previously studied.^16^^,^^26^ In addition, these studies analyse differences between asymptomatic and symptomatic periods, but they do not describe what is visible on individual scans during asymptomatic periods. Our results here, performed in a large series, help determine how sensitive a brain scan visual measure is, at the level of an individual patient, during the diagnosis procedure in KLS. Patients are often referred during the asymptomatic period because performing the FDG-PET/CT during symptomatic periods is challenging, as patients may be unmovable, uncompliant or sleeping during the test.^26^ The sensitivity of abnormal FDG-PET/CT is high, reaching 70% here. This sensitivity increases up to 86% during symptomatic periods (but in *N* = 14 patients). As a comparison, brain ECD scintigraphy yielded a 28.6% sensitivity for visually detecting hypoperfusions in seven boys during asymptomatic KLS periods,^9^ and brain HMPAO scintigraphy yielded a 48% sensitivity for detecting hypoperfusions in 24 patients.^20^ Consequently, PET-FDG/CT seems to be more efficient than HMPAO and ECD scintigraphy at detecting functional abnormalities in KLS brains. Notably, the time since the last episode is not associated here with the presence of hypometabolism (regardless of the affected area), suggesting that these hypometabolisms are trait markers of the disorder. We cannot determine the specificity of the method, as it is not applied here to other neurological and psychiatric disorders that could mimic some KLS symptoms or to an appropriate young healthy control group (not ethical, especially in teenagers). Note, however, that the abnormal metabolism found in bipolar disorder (limbic hypermetabolism and frontal hypometabolism) and in narcolepsy (limbic hypermetabolism) affects different areas than in KLS.^27^^,^^28^

### Hypometabolism in posterior associative cortex

The major finding here is frequent hypometabolism in the posterior associative cortex, which is found in 62.3% of patients with KLS. This location is coherent with the hypoperfusion in the left superior temporal cortices and insula observed in ECD scintigraphy in our 41 previous (and different) asymptomatic patients with KLS versus healthy controls found by group analysis,^13^ but this location is not detected by HMPAO scintigraphy.^20^ This hypometabolism in the posterior associative cortex is associated with female sex, younger age at the time of the FDG-PET/CT, a shorter disease course and a higher number of episodes during the preceding year. The association with the female sex is robust (75% of girls and women had hypometabolism in the posterior associative cortex) and somehow unexpected, as KLS predominantly affects males. Women with KLS have a few clinical differences from men, including more frequent depression and less frequent hypersexuality during episodes as well as a shorter disease course^23^ and a higher risk of emergent psychiatric disorders during asymptomatic periods.^4^ Younger age at the time of the FDG-PET/CT and a shorter disease course (and probably a high number of episodes in the last year, as episodes are usually more frequent during the first years of the disorder) are markers of a recent disorder. This impairment can be graded from hypometabolism restricted to the left temporo-occipital junction, then extended to the rest of the left posterior associative cortex, and then bilaterally. In this case, the larger extension is associated with a more recent disease course (which corresponds to a lower total number of episodes and the amount of time incapacitated). These findings reinforce the idea that hypometabolism and the severity of hypometabolism are markers of a more recent disorder. Based on FDG-PET/CT findings, one may hypothesize that there is brain inflammation at the beginning of the disease (here, within 3 years after onset) that is later attenuated. In this direction, KLS is different from other inflammatory brain diseases, such as multiple sclerosis, as episodes tend to become less severe and less frequent with time and often disappear after 30 years of age.

### Hippocampal hypometabolism

There is hypometabolism in the mesotemporal cortex, including the hippocampus, in almost half of patients with KLS, which is mostly bilateral (21.7%) or affects the left hippocampus (16%). This area is normal in brain perfusion scintigraphy using group analysis in 41 asymptomatic patients with KLS,^13^ but a visual analysis found that 2 of 4 patients with KLS have hypoperfusion in the medial part of the temporal lobe in HMPAO scintigraphy,^2^ as here. The determinants of hippocampal hypometabolism include birth problems, younger age at the time of the FDG-PET, more recent KLS onset (which corresponds to a shorter total number of episodes), and a higher number of episodes in the last year. Interestingly, birth problems are more frequent in patients with KLS than in controls in several large series.^23^^,^^29^ One may wonder whether this brain structure has been damaged during the delivery and if it may be linked to a large intraventricular haemorrhage; however, such haemorrhages are observed in extreme prematurity, which was not the case here. A recent genetic study (performed in most KLS cases in the world, including the patients here) suggests that the risk of developing KLS results from an interaction between a genetic (carrying a TRANK-1 gene mutation) predisposition and birth problems.^30^ As the hippocampus plays a major role in episodic memory, one may be anxious about observing some hypometabolism in this area, especially in teenagers and young adults following academic studies. Indeed, approximately one-third of 120 patients with KLS have residual, mild cognitive problems during asymptomatic periods, including reduced logical reasoning, short-term verbal memory, processing speed, attention and retrieval strategies in verbal memory.^3^ In eight patients with KLS, functional MRI performed during a working memory task indicates that patients have deficient executive and associative networks during rest but are able to compensate them by recruiting different networks during the memory task.^31^ In our series, 134 of 138 patients underwent complete cognitive testing the same day as the FDG-PET. More patients with than without hippocampal hypometabolism have reduced working memory (which uses a frontoparietal network, possibly affected here via hypometabolism in the posterior associative cortex), but surprisingly, they have no more frequent impairment in other cognitive domains (including attention, executive functions and episodic verbal memory). The deficiencies caused by hippocampal hypometabolism may be compensated by other networks in these young brains.

### Prefrontal dorsolateral hypermetabolism

There was visible hypermetabolism in the prefrontal dorsolateral cortex (more often the right than the left part) in one-third of the patients. This hypermetabolism was exceptionally (*N* = 3/48 patients) isolated and mostly (*N* = 45/48) associated with concomitant hypometabolism in the posterior associative cortex and in the hippocampus (more often the left than the right part). Widespread hypermetabolism (in the frontal and temporal cortices, posterior cingulate and precuneus) and no hypometabolism were found in four asymptomatic patients with KLS versus healthy controls using a group analysis in FDG-PET.^16^ These major differences from our study may be due to different (*N* = 138 versus *N* = 4 patients) group sizes and to the method of analysis (visual individual analysis versus group analysis). In our series, hypermetabolism was more frequent at a younger age and when the disease course was shorter, again suggesting, as for hypometabolism, that this anomaly is a marker of recent KLS. One could imagine that this prefrontal hypermetabolism would correlate with more frequent symptoms of the frontal lobe (e.g. hypersexuality or megaphagia) during episodes, but this is not the case in our series and may rather concern the orbitofrontal cortex rather than the prefrontal cortex. Alternatively, prefrontal hypermetabolism during the asymptomatic period could be viewed as a compensatory mechanism for some deficient networks.

### Limitations and perspectives

The main limitation of the study is the absence of a control group of young subjects. This could be useful to determine the specificity of the findings here to the price of irradiating a large group of very young subjects (when FDG-PET/CT is rarely used in other disorders affecting young subjects, i.e. haemopathies). One should note that the FDG-PET/CT was performed in a single expert centre that is highly experienced in KLS, which means that local nuclear medicine physicians may have been trained to detect subtle changes in KLS brain imaging. On the other hand, the study provides reliable measures (e.g. date of first KLS episode is a precise date, because of the sudden aspect of the disease; only untreated patients were analysed to avoid the variability linked to treatments) and homogenous analysis (the same clinicians for diagnosis, the same nuclear medicine physicians for visual analysis and the same neuropsychologist for cognitive tests) in a large group of patients having this extremely rare disorder. Further analyses could be more quantitative using voxel analysis. The predictive role of hypometabolism in determining the evolution of KLS (e.g. are patients with hypometabolism exposed to longer periods of disease, more severe episodes or residual symptoms?) remains to be determined in the future. Longitudinal FDG-PET/CT studies looking at whether hypometabolism disappears when KLS improves or changes with time and disease evolution are needed. Eventually, our study was performed during resting state, which is different from activations during cognitive tests on fMRI as previously performed,^19^^,^^31^ and may explain why we rarely found some thalamic dysfunction. Overall, hypometabolism is frequent on visual analysis of the FDG-PET/CT in KLS, affecting mostly the posterior associative cortex and the hippocampus and is associated with young age and recent (<3 years) onset. Consequently, this information could be useful for clinicians rarely seeing patients with KLS to help them use this trait marker during the diagnosis process.

## Funding

The National Reference Center for Kleine-Levin syndrome is financed in part by a recurrent grant from the French Health Ministry (National Programs on Rare Diseases N#2 and N#3). The promotor of the data collection is Assistance Publique—Hôpitaux de Paris.

## Competing interests

The authors report no competing interest*s*

## Abbreviations

ECD =: ethyl cysteinate dimer;
FDG-PET/CT =: 18F-fluorodeoxyglucose positron emission tomography/computerized tomography scan;
HMPAO =: hexa-methyl-propryl-amineoxime;
KLS =: Kleine–Levin syndrome;
SPECT =: ingle photon emission computed tomography
